# Suffering London

**Published:** 1892-06-25

**Authors:** 


### "SUFFERING LONDON."

### STIRRING ADDRESSES BY SIR ANDREW CLARK AND MR. A. EGMONT HAKE.

A public meeting was held on the 17th inst., at the Mansion House, under the presidency of the LORD MAYOR, and under the auspices of the Metropolitan Hospital Sunday Fund, to hear an address by Mr. A. Egmont Hake, author of "Suffering London."

MR. A. EGMONT HAKE said: My purpose in coming before you to-day is three-fold: first to remind you of the sufferings of our fellow-citizens; secondly, to clear away certain misconceptions which have arisen concerning those institutions whose mission it is to succour the suffering poor; and, thirdly to consider how we may best join hands in support of our hospitals in their courageous battle with disease. The universal interest which is now manifested throughout this great city in the general condition, the fierce struggle, the poignant sufferings of the destitute bears witness to a mighty awakening, to a free and frank recognition of the claims which the unfortunate many must ever have upon the fortunate few. For I think we must all agree that the privations of the people have for some time been a subject of genuine and widespread lamentation. The presence of fabulous wealth side by side with ghastliest poverty; the long continued depression in industry and trade; the bitter complaints against the severity of competition; the constant recurrence of strikes; the exposure of that vile and disgraceful form of slavery, the sweating system; the constant and contradictory cry of over-population and over-production; the devices for depriving this country of its best blood by the agency of emigration; the anxiety to banish the destitute foreigner from our shores—all these circumstances serve to show that the condition of the poorer classes is the foremost question of the day. (Applause.) Mr. Hake went on to say: Let us remember that if we cannot forthwith bring about the amelioration of the people's condition, we can and must strive to lessen those physical sufferings which arise from bodily ailments, and which are made doubly poignant by the presence of poverty and privation. No one will, I believe, charge me with exaggeration when I declare that if the refuges for the sick are permitted to remain as inadequate as they are at present, we shall intensify the physical sufferings of a huge body of the people; we shall increase social misery, and we shall augment the prevailing discontent of the labouring population.

But, besides the responsibility which rests with the classes here represented, with what may be called the flower of the nation, there is another motive which should induce the wealthy to maintain our voluntary hospitals in a state of prosperity—namely, the motive of self-interest. For the advance of medical science, the thorough practice of surgery, the training of nurses, the arrest of epidemics, the development of hygiene—all these advantages, conferred by well-managed hospitals on society, are of as vital importance to the rich as to the poor. (Applause.) In continuance, Mr. Hake said :—Never was there a movement that called for deeper sympathy than the hospital movement, for the hospitals combat one of the most potent causes of poverty and misery—namely, sickness. Realise what it means to the struggling workers, that out of every six applicants five are refused admission simply because the hospitals are short of funds. It means that an appalling number of cases which should be nursed in the hospitals are banished to the people's homes. We are disposed to judge calamities by their effect upon ourselves and upon our particular circle. But let us not forget that circumstances may enormously augment the poignancy of misfortune. To us all sickness and disease are terrible enough, but when they fall upon the struggling poor they should evoke the sympathies of every human heart. For what are the London poor? A huge population of workers fighting bravely, but often vainly, for their lives; shelter or no shelter, a mere lottery; their worldly goods week by week in pawn, and, in their struggle to beat back the demons of penury and starvation, risking existence to win a day. And when sickness comes, and lack of charity has shut the hospital door, a new terror has crept into their lives. And the families that can trace their ruin to a case of illness may be counted by the thousand. For how is it possible for the poor to tend their sick when one-third of the population of this city have barely a pound a week with which to support a whole family? Who, then, among us dare say that the suffering poor have not a powerful claim upon the charity dispensed by the voluntary hospitals? And why are the voluntary hospitals the best and the most trustworthy channels through which to practise indirect charity? Because they owe their existence to the combined efforts of the pick of the community, because they have grown up, have been organised and are administered by means of a system of individual enterprise and individual co-operation, and because such help as we may tender them will pass into good hands and will benefit those who, if bereft of medical aid, must suffer tenfold, and, in many cases, die. Now I hold that our voluntary hospitals would be in a more flourishing condition were it not for certain misconceptions concerning them. For there are certain people who suppose that our hospitals are Government institutions upon which an unlimited demand can be made, and not voluntary institutions supported by charity; there are certain people who forget that hospital [management should not be criticised without technical knowledge, and who seem unaware that rash and irresponsible criticisms of them hampers those citizens who bear the burden of their administration; there are certain people under the impression that the hospitals have a sufficient number of hands, while the fact is that personal services are almost as sorely needed as funds; and there are certain people who make no distinction between voluntary hospitals and rate-supported hospitals, and are apt to condemn the former for the shortcomings of the latter. But, in addition to all this, there is a prevailing prejudice which most seriously militates against contributions to the voluntary hospitals. I mean the prejudice in favour of their socialisation. That our hospitals should be taken over by the Government or the municipality and be supported by the rates is an oft-repeated parrot-cry, the full meaning of which is seldom understood. We all know how such cries become popular. And who are the people who boldly recommend socialisation, and would fain convey the impression that they have mastered the question and have found the solution? I declare that there is not a single argument in favour of the socialisation of our hospitals. But of objections against their socialisation there is a formidable array, of which I will mention a few. To place the hospitals upon the rates would be to subject them to bureaucratic management; would be to drag them down to the low level of State hospitals abroad; would result in a better accommodation for the rich than for the poor; would raise up prejudices against hospital treatment; would degrade hospital patients as the workhouse degrades its inmates now; would bring extra taxation upon employers of labour, and thus increase the number of the unemployed and reduce wages; would deprive the wealthy classes of the high privilege and opportunity of exercising brotherly charity towards their fellow-men; would widen the gulf between the classes, and would pave the way for complete State tyranny. (Applause.) And so, there is only one way of remedying the present state of things. And what is that? Charity—liberal, brotherly, Christian charity. All that is needed is to remember, and to make our friends remember, that there is this great national humanitarian work waiting for us. No matter to what class we may belong, no matter what our means, no matter what our age, we can all easily find a way of taking a part in this glorious work, and of thus enriching our lives. I have no doubt that there are many in this hall who have personally experienced the fact that charity is doubly blest. (Applause.) And so shall we find it a thankful task to help the suffering and the destitute, for the desire for self-sacrifice lies deep in every heart. Civilisation and 2,000 years of Christianity have not smothered, but developed it. Whenever in this country an accident calls for volunteers to risk their lives, the supply is generally in excess of the demand; our soldiers, when required, unhesitatingly give their lives for their country; sea captains sink calmly with their ships when they have seen that their passengers are safe, and only a few weeks ago twenty-seven miners met their death in a burning mine in an attempt to save their comrades. (Applause.) It is such deeds as these that make us proud of our race, and, given the opportunity, I feel sure there will not be any lack of heroism among us Londoners, and this it is that makes me feel that I shall not have appealed to you in vain for that which is perhaps more than heroism—a constant and effective sympathy with the mass of sufferers; a sympathy that must be given without excitement, which will bring forth no applause, no reward beyond the knowledge that you have furthered God's kingdom upon earth. (Loud applause.)

Sir Andrew Clark, who rose to support Mr. Hake, dwelt on the sad accident which happened at the Bishopsgate Station, and drew an affecting picture of the probable condition of the unfortunate sufferers were there no general hospitals open to receive them. As it was, they were taken, he said, at once into bright, airy, healthy wards, the highest surgical skill was immediately put at their service, trained nurses carried out their instructions, and everything was done which could have been done for the greatest person in the land. (Applause.) No one pretending to any of the higher feelings of humanity could doubt for a momentthat those things which were done, and which are being yet done for these sufferers, were not great things worthy to be done—(applause)—that they were not the very things which make this life of ours sometimes noble, and which, furthermore, make the sacrifice which it entails a matter of everlasting good. (Applause.) But this is not all that happened, and is happening. For instance, the surgeons who are attending upon these cases are acquiring additional experience and skill, the students standing by are being taught, the nurses are receiving extra training, the fountains of human sympathy are being opened between the sufferer and the server, and the knowledge thus acquired within the hospital walls cannot be kept within them but passes out and enters into every house in the land, conveying to every human soul therein." He further pointed out the terrible condition of London if there were no hospitals for infectious diseases, and no means of alleviation by skilled care and proper surroundings of the numerous inevitable accidents incurred by the working population. One of the previous speakers asked you to endeavour to raise £50,000 this year, and the other £100,000; well, I will go between the two and ask you to raise £75,000 this year—(laughter) — although that will not really be sufficient. (Hear, hear.) In moving the resolution I have to move, I earnestly request that you will yourselves give, and that you will use all the influence you possess to make others give also. (Hear, hear.) There are many reasons for giving to this fund, and what is of more importance I regard those reasons as unanswerable. First of all then this fund offers an opportunity of collecting in the cheapest way possible small sums of money that probably would otherwise never reach the hospitals at all. It catches them on the way, and it enables the givers of these small sums to take a pride therein. Secondly, it gives a personal interest to all those who contribute on the Sunday to this great work, and it fosters a feeling (which I hope will be a growing one) of brotherhood of charity in sustaining these great hospitals. Thirdly, it opens a way for making small contributions express much sympathy. The small contribution that the widow might give on Sunday enables her to open her heart to all the charities which she has any feeling for without the humiliation of giving a penny here and a halfpenny there. Lastly, and this is a greater reason than all, the Council of the fund, in keeping a vigilant eye upon the working and the spending of all the hospitals—for it controls its disbursements accordingly—enables itself to exercise not only a salutary, but a very powerful influence on the management of these hospitals. I may sum up now and say in short that by supporting this fund you enable the highest work to be done in the most economical manner. (Applause.) It may be said by an objector: "Times are hard, business is bad, expenses are increasing, and I do not see how I am with any comfort to give more than I am giving." Admitting all this is true, that times are hard, business is bad, and expenses are increasing, I venture to say that those are just the very circumstances in which you ought to exercise your charity. Those are just the very circumstances which touch your charity with fire from heaven, and which transfigures your sacrifice makes, it divine.

The Rev. Prebendary A. Wilmot, who seconded Sir Andrew Clark, remarked that as a minister of religion and as one who thereby came daily into contact with the sufferings and privations of the poor, he would have found his position almost intolerable and his work depressing had it not been for the existence of the hospitals.

Mr. Burdett, in supporting, dwelt on the importance of personal service to the hospitals, and said, did they but get this in an adequate quantity from the citizens of London, it would be almost unnecessary to have a Hospital Sunday Fund at all, as the hospital exchequers would then be full. He especially appealed to the editors of the metropolitan press to co-operate with the clergy and ministers of religion in the future to a greater degree, whilst gratefully acknowledging their services to the Sunday Fund in the past. When, some years ago, the Hospital Sunday Fund fell off very considerably, the editors responded to the appeal made to them by giving space in their columns each day throughout the week preceding Hospital Sunday, and this he appealed to them to do again. If they did that, he believed it would result in an increased contribution of something like £25,000 a year.

Sir Sydney Waterlow, Bart., proposed a vote of thanks to the Lord Mayor, seconded by the Rev. Dr. Kennedy.

The Lord Mayor, in returning thanks, urged his hearers not to be content with merely contributing, but to take a more active personal interest in the hospitals of London generally, and to try and interest their friends in the same way.

